# Data-Driven Prediction and Uncertainty Quantification of Process Parameters for Directed Energy Deposition

**DOI:** 10.3390/ma16237308

**Published:** 2023-11-24

**Authors:** Florian Hermann, Andreas Michalowski, Tim Brünnette, Peter Reimann, Sabrina Vogt, Thomas Graf

**Affiliations:** 1 Graduate School of Excellence Advanced Manufacturing Engineering (GSaME), University of Stuttgart, Nobelstraße 12, 70569 Stuttgart, Germany; andreas.michalowski@ifsw.uni-stuttgart.de (A.M.); peter.reimann@gsame.uni-stuttgart.de (P.R.); thomas.graf@ifsw.uni-stuttgart.de (T.G.); 2 TRUMPF Laser- und Systemtechnik GmbH, Johann-Maus-Straße 2, 71254 Ditzingen, Germany; 3 Institut für Strahlwerkzeuge (IFSW), University of Stuttgart, Pfaffenwaldring 43, 70569 Stuttgart, Germany; 4 Institut für Wasser- und Umweltsystemmodellierung (IWS), University of Stuttgart, Pfaffenwaldring 5a, 70569 Stuttgart, Germany; tim.bruennette@iws.uni-stuttgart.de

**Keywords:** machine learning, Gaussian Process Regression, directed energy deposition, single track geometry, uncertainty quantification, user-centric decision making, expert knowledge

## Abstract

Laser-based directed energy deposition using metal powder (DED-LB/M) offers great potential for a flexible production mainly defined by software. To exploit this potential, knowledge of the process parameters required to achieve a specific track geometry is essential. Existing analytical, numerical, and machine-learning approaches, however, are not yet able to predict the process parameters in a satisfactory way. A trial-&-error approach is therefore usually applied to find the best process parameters. This paper presents a novel user-centric decision-making workflow, in which several combinations of process parameters that are most likely to yield the desired track geometry are proposed to the user. For this purpose, a Gaussian Process Regression (GPR) model, which has the advantage of including uncertainty quantification (UQ), was trained with experimental data to predict the geometry of single DED tracks based on the process parameters. The inherent UQ of the GPR together with the expert knowledge of the user can subsequently be leveraged for the inverse question of finding the best sets of process parameters by minimizing the expected squared deviation between target and actual track geometry. The GPR was trained and validated with a total of 379 cross sections of single tracks and the benefit of the workflow is demonstrated by two exemplary use cases.

## 1. Introduction

Manufacturing companies face the challenge of ever shorter development and product life cycles and individualized products [1,2,3]. Software-defined manufacturing is an approach that enables flexible and reconfigurable systems and is therefore able to handle these challenges [4]. The successful implementation of software-defined manufacturing requires production systems that are as flexible and universal as possible [5] and that are sufficiently defined via software so that they can flexibly adapt to changing specifications [6,7]. Laser-based directed energy deposition with metal powder (DED-LB/M) offers such a flexible process, as it can be used for coating, welding, repairing and additive manufacturing without major change in hardware [8,9,10]. To weld single DED tracks, which are the basis of all mentioned applications, powder is transported to the process zone and the laser beam melts both powder and workpiece leading to a metallurgic bonding [11,12,13]. The geometry of the DED tracks and the corresponding height of the produced layers are influenced by the process parameters such as velocity *v*, laser power *P*, powder flow rate $\dot{m}$ and the diameter $d_{L}$ of the laser beam on the surface of the workpiece. These parameters are all specified and adjusted via software. However, finding suitable process parameters to achieve the required track geometry for each application is currently a highly manual process relying on the individual process knowledge of the operator. Defining the geometry and process parameters in the software layer without performing prior experiments may yet offer a promising step towards software-defined manufacturing. Therefore, models that enable the prediction of the process parameters that yield the desired track geometry are essential for implementing a software-defined workflow.

Physics-based models provide valuable information about the formation of single tracks in DED. Ahsan and Pinkerton [14], for example, propose an analytical-numerical model to predict the geometry of single tracks, El Cheikh et al. [15] analytically describe the geometry of single DED tracks, Gao et al. [16] established a three-dimensional numerical model to predict the single track geometry and temperature distribution for single-tracks, Huang et al. [17] developed a physics-based process model for the prediction of the geometry of single tracks and multi-layer deposition and Zhang et al. [18] developed a three dimensional transient model for evolving temperature fields of thin walls. Despite their undeniable added value, none of the aforementioned models can represent the full complexity of the process. For instance, thermophysical properties are assumed to be constant in Ahsan and Pinkerton [14] and Huang et al. [17], heat convection is neglected in Huang et al. [17], the influence of molten pool fluid and the heat loss caused by vaporization of powder is ignored in Gao et al. [16], the heat that is incorporated into the melt-pool by the powder is neglected and assumptions about the catch efficiency of the powder are made in Zhang et al. [18], the absorption coefficient is determined experimentally in El Cheikh et al. [15] and some input values for the simulation such as the intensity profile of the laser in Gao et al. [16] are prone to some uncertainty. That is why physics-based models lose predictive accuracy in consideration of the process variability [19].

In recent years, data-driven models are becoming increasingly popular for performing such tasks as they are less computationally expensive and do not require that assumptions be made about the underlying physical process [20]. Hereby, deterministic models such as artificial neural networks [21,22,23,24,25,26,27,28] or Regression trees (RT) [24,29] are for example applied to predict the track geometry as a function of the process parameters in DED. However, deterministic models cannot provide uncertainty quantification (UQ), which is crucial for reliable additive manufacturing due to the various sources of uncertainties in additive manufacturing [30,31,32,33]. Probabilistic machine learning models such as Gaussian Process Regression (GPR) [34,35] can account for this UQ and have been applied in laser powder bed fusion (LPBF) to predict the melt pool geometry [36,37,38,39,40,41] or in DED to predict the mechanical properties [42], the component height [43], the geometry of single tracks [44,45], or melt pool geometry [46,47] based on the process parameters. The inverse problem, i.e., the determination of a suitable process to produce the desired track geometry, can principally be solved by combining the regression model with an optimization algorithm. In this context, GPR may be combined with a global optimization algorithm, for example, to minimize distortion in fused filament fabrication (FFF) [48], to optimize the microstructure in electron beam melting (EBM) [32], to reduce the surface roughness and the geometric deviation in LPBF, or to optimize the parameters with respect to the mechanical properties in DED [49]. Mondal et al. [50] trained a GPR model with simulation data for predicting the melt pool geometry as a function of laser power *P* and velocity *v*, and performed a Bayesian optimization to determine the optimal parameter combination to keep the geometry of the melt pool at a suitable value.

However, with an increasing number of considered process parameters, different sets of parameters may lead to the same processing results. Therefore, we include the prediction uncertainty of each combination of process parameters as well as expert knowledge when selecting the best parameters to manufacture a desired track geometry. Thus, this paper presents a novel workflow to select multiple parameter combinations that are most likely to yield the desired track geometry in DED. This is achieved by combining a GPR-model with an optimization algorithm that identifies multiple suitable sets of process parameters based both on the deviation from the targeted geometry of the single tracks as well as on the uncertainty of the prediction. The consideration of several possible parameter combinations allows the user to select the process parameters that best suit the application in question and make an informed decision on how to manufacture the component. Section 2 introduces the workflow on a general level, highlighting the interaction between the building blocks. Section 3.1 describes how the experimental data are obtained, while Section 3.2 introduces the GPR model. The data are subsequently used in Section 4 to validate the workflow, give exemplary applications, and discuss quantitative results apparent in the given DED process.

## 2. Prediction Workflow

**Figure 1:**
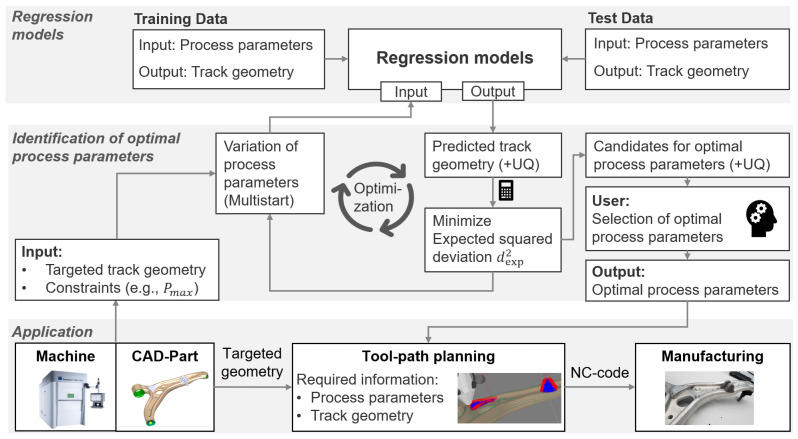
Workflow for finding optimal process parameters.

As schematically shown in Figure 1, the workflow to determine suitable processing parameters consists of three main elements:

- Regression models
- Identification of optimal process parameters
- Application

The manufacturing of the component takes place in the application layer that is displayed at the bottom of Figure 1. Based on the requirements from the application, the user defines the targeted geometry of a single track and the constraints on the process parameters. The targeted track geometry results from the geometry of the component (usually a CAD-part), and the constraints on the process parameters are mostly given by the limits of the given machine. In return, the application layer requires information about the optimal process parameters that lead to the targeted track geometry in order to be able to perform the toolpath planning. The toolpaths and process parameters are stored in a numerical control (NC) code that is readable by the machine and that enables manufacturing of the component. The probabilistic regression models that are displayed at the top of Figure 1 are essential to identify the required process parameters. These models predict the geometry of single tracks based on the process parameters and offer an uncertainty quantification (UQ) of the prediction. Parts of the available data, which are described in Section 3.1, were used to train the models and the rest of the data were used to test the performance of the models.

To answer the inverse question of finding optimal process parameters for a given targeted geometry, we implemented the optimization workflow that is displayed in the middle of Figure 1. Finding the optimal set of process parameters to achieve a specific track geometry may be a trade-off between accuracy and uncertainty. This choice depends on whether one prefers a precise prediction with tight tolerances but a high uncertainty or a less accurate prediction that is fulfilled with a higher probability. While both accuracy and uncertainty could be kept as separate optimization goals, we incorporate them into a single measure of optimality. The expected squared deviation $d_{\exp}^{2}$ between the predicted values y(x) of a geometric property (such as height, width or melting depth) of the track obtained with a set **x** of processing parameters (e.g. laser power, beam diameter, velocity and powder flow rate) and the targeted value *z* of each geometric property can be expressed by

(1) $$d_{\exp}^{2}\left(x\right)=E\left[||y\left(x\right)-{z||}^{2}\right]=\mathrm{Var}\left[y\left(x\right)\right]+\left|\right|\bar{y}\left(x\right)-z{\left|\right|}^{2}.$$

Using the approach of GPR, the predicted values y(x) are subject to a Gaussian probability distribution with the expected value $\bar{y}\left(x\right)$. The value of $d_{\exp}^{2}\left(x\right)$ is then minimized in order to identify the optimal process parameters. As we want to identify multiple sets of process parameters at different parameter ranges, we use Newton optimization in combination with a multistart strategy. The step size Δx of the Newton algorithm is limited to ensure that the algorithm converges to the closest local minimum and the multistart strategy augments the probability that all relevant local minima are identified during the optimization. The process parameters x are varied within the constraints on the search space as given by the user (depending on the used machine) with a step size that is varied based on the hyperparameters of the GPR models. The process parameters corresponding to the identified local minima are subsequently delivered to the application layer, which allows the parameters that best suit the application to be selected.

**Figure 2:**
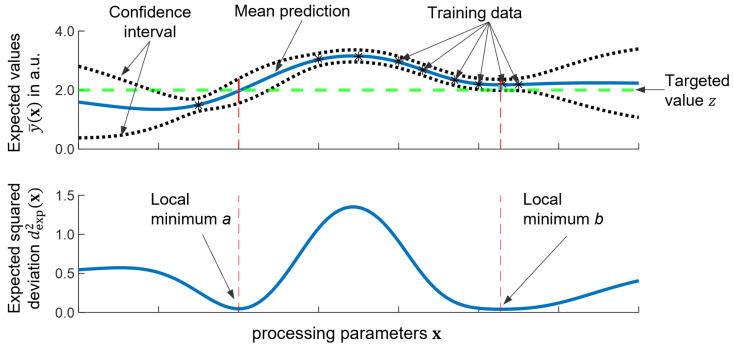
Fictional illustration of the approach. (**Top**): expected values $\bar{y}\left(x\right)$ (blue), variation of y(x) as given by the 95% confidence interval (black dotted) and targeted value *z* (green dashed). (**Bottom**): expected squared deviation $d_{\exp}^{2}\left(x\right)$ in relation to the targeted value *z*.

The principle of the identification of the most promising processing parameters and the involved quantities are illustrated by Figure 2.

The stars in the upper diagram represent the training data, which are obtained from experiments. The GPR then yields the expected values $\bar{y}\left(x\right)$ (blue, top graph) and the variation of y(x), as represented by the 95% confidence interval (black dotted). The green dashed line represents the targeted value *z*. The red dashed lines mark the local minima of the expected squared deviation $d_{\exp}^{2}$ (**x**) defining the most promising sets of processing parameters $x_{a,b}$. At the local minimum *a*, the expected value $\bar{y}\left(x_{a}\right)$ equals the targeted value *z*, but the variance of $y(x_{a})$ is larger than at the local minimum *b*, where the expected value $\bar{y}\left(x_{b}\right)$, however, does not correspond exactly to the desired value *z*. Hence, although the certainty of the predicted value $\bar{y}\left(x_{b}\right)$ is higher at the local minimum *b*, the application of the corresponding processing parameters $x_{b}$ is expected to result in a value $y(x_{b})$ slightly larger than the target *z*. Conversely, this means that the expected value $\bar{y}\left(x\right)$ at the local minimum *a* is more accurate but has a higher uncertainty compared to the one obtained at the minimum *b*. Both minima are, however, of similar quality with respect to the expected squared deviation $d_{\exp}^{2}\left(x\right)$. Depending on whether uncertainty or accuracy is more important and depending on which of the processing parameters better suit the application in question, the user may either select local minimum *a* or *b*. The choice of a local optimization with multistart instead of a global optimization such as Bayesian optimization or Upper Confidence Bound (UCB) enables the identification of multiple sets of suitable process parameters at different parameter ranges and allows the user to select the set of parameters that best suits the application in question.

## 3. Materials and Methods

To apply the described workflow, the regression models were trained with and tested on experimental results. Section 3.1 describes the experimental set-up and Section 3.2 describes the details of the regression models and how they were trained.

### 3.1. Experimental Data

**Figure 3:**
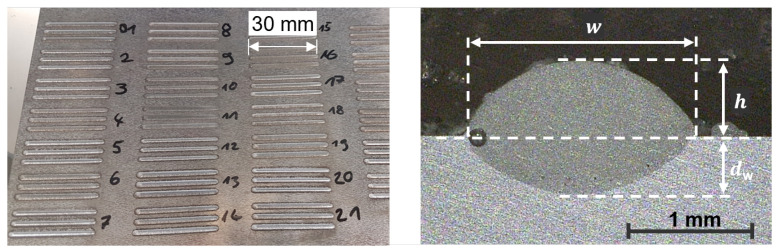
Picture of multiple separate DED tracks (**left**) and microscopical image of a cross section of a single DED track (**right**).

To collect the necessary data for the training of the regression models for the different geometrical features of the DED welding tracks, a total of 379 single tracks were produced on a 10 mm thick plate of AlMg3 using different process parameters. Some of the tracks are exemplarily shown on the left in Figure 3. The DED-LB/M was performed on the five-axis laser machine TruLaser Cell 3000 using the 4 kW disk laser TruDisk4001 with a wavelength of 1030 nm and a beam parameter product of 4 mm × mrad, a laser light cable with a diameter of 100 μm and the optics focusLine Professional all from TRUMPF Laser- und Systemtechnik GmbH, Ditzingen, Germany. The AlSi10Mg powder from Carpenter Additive (CA), Widnes, UK, with particle diameters ranging from 45 to 107 μm, was fed using a vibratory feeder from Medicoat AG, Mägenwil, Switzerland and a helium gas flow with a flow rate of 10 L/min. The multijet nozzle from TRUMPF Laser- und Systemtechnik GmbH, with seven jets arranged coaxially around the laser beam was used as a powder nozzle and Argon with a flow rate of 12 L/min used to shield the process zone from the atmosphere. The powder was melted by a defocused laser beam with a focus diameter of 200 μm and a variable diameter $d_{L}$ on the surface of the substrat. Cross-sections of the DED tracks were prepared by cutting, grinding, polishing and etching with a water solution containing 10% sodium hydroxide.

The depth $d_{w}$, the width *w* and the height *h* of the tracks were measured from the resulting cross-sections, as displayed on the right in Figure 3, by means of an optical microscope. The laser power *P*, the mass supply rate $\dot{m}$ of the powder, the diameter $d_{L}$ of the laser beam on the surface, and the velocity *v* have a significant influence on the geometry of the resulting track and were therefore varied over a wide range and in variable steps: *P* between 1 and 4 kW in 16 steps, $\dot{m}$ between 0 and 42 g/min in 21 steps, $d_{L}$ between 1 and 2 mm in 4 steps and *v* between 0.75 and 20 m/min in 14 steps. Each combination of parameters was repeated at least three times, resulting in a total of 379 single tracks.

### 3.2. Training of Regression Models

Since the identification of optimal process parameters described in Section 2 is based on the probability of producing the targeted track geometry, a prediction model with built-in uncertainty quantification is required. The specific mathematical tool employed in this work is Gaussian Process Regression (GPR), which is capable of quantifying the uncertainty. We trained one regression model to predict the width *w*, one for predicting the height *h* and one for the prediction of the depth $d_{w}$ of a single track. The models use the laser power *P*, the mass supply rate $\dot{m}$, the velocity *v* and the beam diameter $d_{L}$ as the relevant processing parameters. A fraction of 80% of the acquired experimental data was randomly selected to train the models using k-fold cross-validation with five folds. The use of k-fold cross-validation enables a more robust model, which is less sensitive to the sampling of the data and the specification of the prior [51]. The remaining 20% of the data were used to test the performance of the data-driven models. All the repetitions of the experiments with the same set of processing parameters were kept in the same control group to make sure that the test data set only contained parameter combinations that the models were not trained on before. We integrated linear basis functions into our models because we assume that a linear trend of the mean-function can be continued to some extent when extrapolated at the boundaries of our experimental domain. For this, we used the same approach as described in ([34], Section 2.7) where the dependant variable y(x) of the regression model, which is the predicted track geometry in our application, is modelled by

(2) $$\begin{array}{c} y\left(x\right)=f\left(x\right)+q{\left(x\right)}^{T}\beta . \end{array}$$

Here, q(x) contains the linear basis functions and β the corresponding coefficients, which are determined from the data. The function f(x) denotes the prediction of a Gaussian process at the parameter vector x with a zero mean-function and a squared exponential kernel *k*, which is defined by

(3) $$\begin{array}{c} k\left(x_{i},x_{j}\right)=\sigma_{f}^{2}\;\exp\left[-\frac{1}{2}\sum_{m=1}^{d}\frac{{\left(x_{j,m}-x_{i,m}\right)}^{2}}{{\left(l_{m}\right)}^{2}}\right] \end{array}$$

for two input vectors $x_{i}$ and $x_{j}$ and their elements $x_{i,m}$ and $x_{j,m}$ in the dimension *m*. For our application the input vector x contains the processing parameters and has d=4 dimensions (*P*, $\dot{m}$, $d_{L}$, *v*). The hyperparameters of the kernel *k*, i.e., the length scales $l_{m}$ and the signal variance $\sigma_{f}^{2}$ are determined from the data. Homoscedastic noise is assumed. Therefore, the corresponding covariance matrix *C* is defined by

(4) $$\begin{array}{c} C_{ij}=k\left(x_{i},x_{j}\right)+\sigma^{2}\delta_{ij}, \end{array}$$

where $\delta_{ij}$ is the Kronecker delta and $\sigma^{2}$ is the noise variance. The predicted mean $\bar{f}\left(x\right)$ and the variance Var[f(x)] of the prediction are calculated by

(5) $$\begin{array}{cc} \bar{f}\left(x\right) & =k^{T}C^{-1}t \end{array}$$

(6) $$\begin{array}{cc} \mathrm{Var}[f(x)] & =c-k^{T}C^{-1}k, \end{array}$$

where the scalar c=k(x,x), the vector k has the elements $k(x_{n},x)$ for n=1,…,N and the vector t contains the measured target values at the input points $x_{n}$. *N* represents the number of data points used to train the models. The data are normalized in the input space and in the output space before training the models.

## 4. Results and Discussion

To show that the workflow described above can be successfully applied to predict the processing parameters required to produce the desired geometry of single tracks in DED-LB/M, we applied our workflow and regression models to the previously described test data. The predictive quality of the models as a function of the process parameters is discussed in Section 4.1. Section 4.2 is then devoted to the inverse problem of finding optimal process parameters. The workflow is found to yield plausible results and we show how the user can interact and profit in realistic scenarios.

### 4.1. Analysis of Regression Models

For each quantity of interest, i.e., track-width, track-height, and track-depth, a separate Gaussian process was trained with the input parameters laser power *P*, mass supply rate $\dot{m}$ of the powder, laser beam diameter $d_{L}$ and velocity *v*. The resulting length scales $l_{m}$, which are determined via automated relevance determination (ARD), show the influence of the input parameters on the respective output quantity, whereby a small length scale indicates possible changes in output even for small changes in input. The coefficients β of the linear model in Equation (7) provide information about the offset and the linear trends regarding the influence of the input parameters on the respective output quantity. The mean-absolute-error (MAE) between the mean value $\bar{y}\left(x\right)$ of the prediction and the corresponding value $y_{\mathrm{test}}\left(x\right)$ of the test data set as well as the coefficient of determination $R^{2}$, which is calculated by

(7) $$\begin{array}{c} R^{2}=1-\frac{\sum {\left(y_{\mathrm{test}}\left(x\right)-\bar{y}\left(x\right)\right)}^{2}}{\sum {\left(y_{\mathrm{test}}\left(x\right)-{\bar{y}}_{\mathrm{test}}\right)}^{2}}\;, \end{array}$$

were used as a measure to evaluate the accuracy. Hereby, ${\bar{y}}_{\mathrm{test}}$ is the average value of the test data set. The resulting numerical values are summarized in Table 1. The expected prediction errors that arise for track-width *w*, track-height *h* and track-depth $d_{w}$ are tolerable for most applications. The two values $R^{2}$ and MAE only consider the mean prediction of the models and therefore enable comparison to other deterministic models. However, in the following, we present capabilities that are exclusive to the probabilistic paradigm.

**Figure 4:**
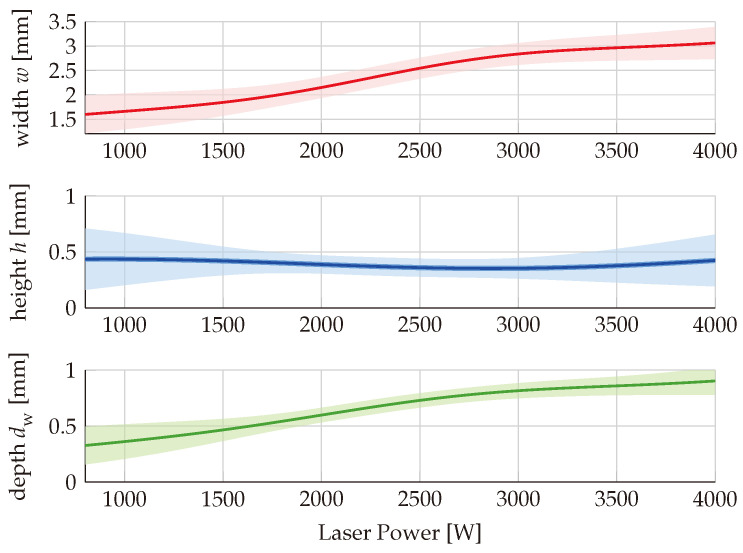
Dependence of the predicted track geometry on the applied laser power for v= 2 m/min, $d_{L}\;=$ 2 mm and $\dot{m}=$ 2 g/min.

For the discussion of the influence of the process parameters on the processing result, Figure 4 exemplarily shows the predicted dependence of the width (in red), the height (in blue), and the depth (in green) of the tracks on the laser power. All other parameters are kept constant: v= 2 m/min, $d_{L}=$ 2 mm and $\dot{m}=$ 2 g/min. The pale-colored areas represent the 95 % confidence intervals around the predicted values (solid lines).

The track width and track depth both increase steadily with increasing laser power. This can be explained by an increase of the volume of the melt pool. The height of the track does not change significantly. The fact that the product of the track width and the height increases despite the constant powder flow rate indicates that increasing the laser power yields an increased powder efficiency. The laser power that was applied in the training data mostly ranged between 2000 W and 3400 W. This is why the uncertainties of the predictions are lower in this range of laser power and increase significantly when the predictions are made for laser powers above or below this range.

### 4.2. Identification of Optimal Process Parameters

#### 4.2.1. Multiple Local Minima

The proposed optimization approach is able to find multiple sets of optimal process parameters corresponding to the local minima of the expected squared deviation. This allows the user to select the parameter combination that best fits a given application.

To illustrate how our optimization routine identifies these local minima, we exemplarily defined a targeted geometry with w= 2.5 mm, h= 0.45 mm and $d_{w}=$ 0.6 mm. In the optimization procedure the process parameters were varied with equidistant steps that correspond to one non-standardized length scale of the GPR process, i.e., ΔP=936 W, $\Delta d_{L}=0.33\;\mathrm{mm}$ and Δv=0.35 m/min. The mass supply rate $\dot{m}$ of the powder was adapted in a way that the mass per distance remains constant at 2.1 g/m. These initial parameters yield the different local optima listed in Table 2. They are sorted from the lowest to highest expected squared deviation and are also provided to the user in this way.

**Figure 5:**
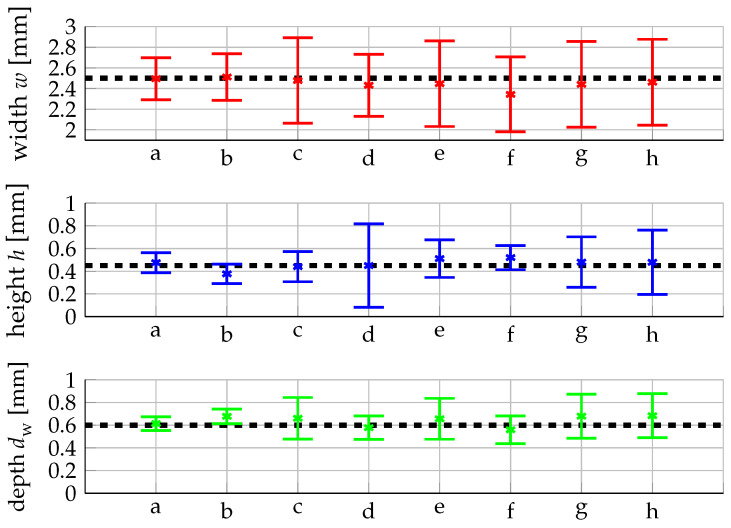
Predicted track geometry for the different local minima listed in Table 2.

The corresponding predicted track geometry is shown in Figure 5 together with the 95% confidence interval. The black dashed lines represent the targeted values. It is evident that there is more than one set of process parameters leading to the targeted geometry and that the combination of the multistart with our optimization algorithm is able to identify these suitable sets of process parameters. A comparison with a gridsearch optimization revealed that our optimization identifies all interesting local minima.

By proposing several sets of suitable processing parameters, we provide the user with sufficient information to asses which process parameters best suit a given application. For our exemplary target geometry, local optimum a, cf. Figure 5, exhibits the smallest expected squared deviation and the expected depth and height are significantly closer to the targeted value as compared to local optimum b. When the height and the depth of a track are critical for the application, the user will most likely opt for the parameter set a. If multiple local minima are of similar quality the user may also consider further criteria for the selection of process parameters based on his or her expert knowledge: higher velocities may be preferred for economic reasons or lower laser power may be preferred when dealing with heat sensitive parts.

#### 4.2.2. Optimal Process Parameters at Different Velocities

In many industrial applications, certain cycle times and thus velocities have to be achieved in order to be economical [52]. In the following, we therefore demonstrate how our workflow can be used to find the optimal processing parameters at a given velocity using an exemplary targeted track geometry of *w* = 2 mm, *h* = 0.45 mm and $d_{w}$ = 0.5 mm and perform the optimization for ten different fixed velocities from 2 to 20 m/min. The parameters that are most likely to yield the targeted geometry at the given velocity are listed in Table 3.

**Figure 6:**
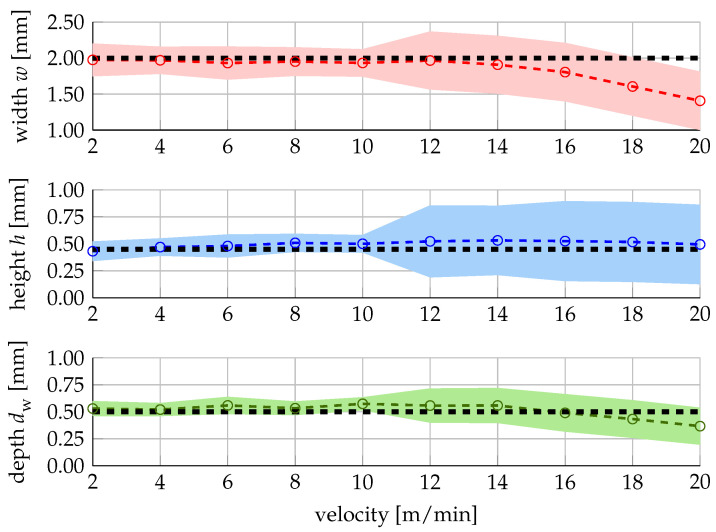
Predicted track geometry for the optimal process parameters listed in Table 3.

The mean of the predicted geometries (coloured circles connected by dashed lines), including their 95% confidence interval (pale-couloured areas) for these parameters, are displayed in Figure 6. The targeted geometry is indicated by the black dashed lines. The predictions match the target reasonably well with an expected squared deviation of less than 0.03 mm^2^ up to a velocitiy of 10 m/min. For higher velocities, the prediction quality deteriorates both in closeness to the targeted value and certainty for all three geometrical features, cf. Figure 6. The increased uncertainty observed for all three geometric characteristics at feedrates above 10 m/min is due to the low number of training data in the range of these identified parameter combinations. The mean of the predicted track width deviates downwards from the targeted value for feedrates above 12 m/min and the mean of the predicted track depth for velocities above 16 m/min. These observations are consistent with those of [53], where it was observed that a maximum laser power of 4 kW is insufficient to weld tracks with a width of 2 mm up to velocities of 20 m/min and that both the maximum track width and track depth decrease for velocities above 10 m/min due to the limited laser power. The information about the processing parameters, the uncertainty of the prediction, and the deviation from the targeted geometry enables a user to make an informed decision regarding the economic stipulations and quality requirements.

## 5. Conclusions

In conclusion, it was shown that the combination of an optimization workflow and expert knowledge with probabilistic regression models such as GPR enables us to predict the process parameters needed to achieve a specific track geometry for laser-based directed energy deposition using metal powder (DED-LB/M). The validation with a large number of individual tracks revealed a good agreement between the test data and the predictions of the regression models. The usefulness and applicability of the proposed workflow for a user to make an informed decision on optimal process parameters as well as for receiving optimal process parameters at different velocities has been demonstrated with two exemplary targeted geometries of a single track. The proposed workflow thus provides a promising step towards software-defined manufacturing.

Even though the potential of that workflow has been shown, further investigation may be undertaken. The models are so far only applied to isolated welding tracks on a plane sample. A generalization of the models towards more complex geometric scenarios may enable a much wider range of uses. This incorporates many new challenges, such as the detection and the addition of other relevant parameters and effects such as heat accumulation. The latter challenge may be tackled by the integration of uncertainty-aware temperature predictions, as proposed by Sideris et al. [20]. Furthermore, to get closer to an efficient industrial application, the generation of the data for this data-based model can be automated to make it less time-consuming. The combination of automated and optimized selection of parameters with automated data acquisition allows for quick training of the model and a rapid adaption of the workflow to new circumstances such as the use of different alloys. Bayesian optimization or upper-confidence bound (UCB), both of which merge the mean and variance of a prediction, may be used in order to design a data-efficient adaptive experimental design. One possibility to adapt the model to new environments is to train only the deviation from the old model instead of training the model from scratch.

## Figures and Tables

**Table 1:** Optimal hyperparameters and accuracy of the GPR models.

	σ	$l_{m}$ per Predictor (*P*/$\dot{m}$/$d_{L}$/*v*)	*β*: Coefficients of Linear Basis (1/*P*/$\dot{m}$/$d_{L}$/*v*)	$R^{2}$	MAE
*w*	0.08	1.05/0.03/1.05/0.15	[2.06/0.31/0.08/0.09/−0.65]	0.89	0.11 mm
*h*	0.04	1.62/0.25/1.95/0.05	[0.53/0.03/0.45/−0.01/−0.48]	0.88	0.04 mm
$d_{w}$	0.02	1.17/0.04/1.90/0.17	[0.52/0.13/−0.05/0.04/−0.13]	0.91	0.04 mm

**Table 2:** Identified process parameters when searching for local minima of the expected squared deviation.

No.	*P* [W]	$\dot{m}$ [g/min]	$d_{L}$ [mm]	*v* [m/min]	$d_{\exp}^{2}\;\left[{\mathrm{mm}}^{2}\right]$
a	2836	3.0	1.3	1.5	0.014
b	2815	3.3	1.8	2.0	0.027
c	2669	1.6	1.6	1.0	0.060
d	3399	3.1	1.0	2.3	0.064
e	2173	2.1	2.3	1.0	0.071
f	2173	2.0	1.5	1.0	0.076
g	3054	7.3	2.1	4.0	0.082
h	2965	7.3	2.3	3.9	0.083

**Table 3:** Optimal process parameters with lowest expected squared deviation at different velocities.

*v* [m/min]	*P* [W]	$\dot{m}$ [g/min]	$d_{L}$ [mm]	$d_{\exp}^{2}\;\left[{\mathrm{mm}}^{2}\right]$
2	1856	3.2	2.0	0.018
4	2188	8.4	2.0	0.014
6	2684	12.6	2.0	0.028
8	3329	16.8	1.1	0.020
10	3867	21.0	2.0	0.025
12	3647	25.1	2.8	0.087
14	4000	29.2	2.7	0.095
16	4000	33.6	3.0	0.130
18	4000	37.7	3.0	0.251
20	4000	42	3.0	0.457

## Data Availability

The data presented in this study are available on request from the corresponding author.

## Abbreviations

The following abbreviations are used in this manuscript:

GPR	Gaussian Process Regression
RT	Regression tree
UQ	Uncertainty quantification
DED	Directed energy deposition
DED-LB/M	Laser-based directed energy deposition using metal powder
LPBF	Laser powder bed fusion
FFF	Fused filament fabrication
EBM	Electron beam melting
CAD	Computer aided design
ARD	Automatic relevance determination
MAE	Mean absolute error
*v*	Velocity
*P*	Laser power
$\dot{m}$	Powder flow rate
$d_{L}$	Laser beam diameter on the surface of the substrate
$d_{\exp}^{2}$	Expected squared deviation between prediction and target value
*z*	Target value of geometry characteristic
y(x)	Prediction performed with the GPR model
$d_{w}$	Depth of of a single DED track
*w*	Depth of a single DED track
*h*	Height of a single DED track
$R^{2}$	Coefficient of determination
q(x)	Vector with linear basis functions
β	Coefficients of linear basis
*k*	Kernel of GPR model
$l_{m}$	Length scale
$\sigma_{f}^{2}$	Signal variance
$\delta_{ij}$	Kronecker delta
$y_{\mathrm{test}}$	Measured values in the test data set
${\bar{y}}_{\mathrm{test}}$	Mean value of $y_{\mathrm{test}}$
